# Early-start antiplatelet therapy after operation in patients with spontaneous intracerebral hemorrhage and high risk of ischemic events (E-start): Protocol for a multi-centered, prospective, open-label, blinded endpoint randomized controlled trial

**DOI:** 10.3389/fnagi.2022.1020224

**Published:** 2022-11-23

**Authors:** Kaiwen Wang, Shaohua Mo, Qingyuan Liu, Jun Pu, Xiaobin Huang, Dezhi Kang, Fixin Lin, Dewei Zou, Xinguo Sun, Jinrui Ren, Xianzeng Tong, Jiangan Li, Rustam Al-Shahi Salman, Nuochuan Wang, Shuaiwei Guo, Yang Liu, Yanan Zhang, Xiong Li, Jun Wu, Shuo Wang

**Affiliations:** ^1^Department of Neurosurgery, Beijing Tiantan Hospital, Capital Medical University, Beijing, China; ^2^China National Clinical Research Center for Neurological Diseases, Beijing, China; ^3^Department of Neurosurgery, The Second Affiliated Hospital, Kunming Medical University, Kunming, China; ^4^Department of Neurosurgery, The First Affiliated Hospital of Fujian Medical University, Fujian Medical University, Fuzhou, China; ^5^Department of Neurosurgery, Chongqing General Hospital, University of Chinese Academy of Sciences, Chongqing, China; ^6^Department of Neurosurgery, Binzhou People's Hospital, Binzhou, China; ^7^Department of Neurosurgery, The People's Hospital of Shanxi Province, Taiyuan, China; ^8^Department of Neurosurgery, Beijing Friendship Hospital, Capital Medical University, Beijing, China; ^9^Department of Neurosurgery, Affiliated Wuxi No. 2 Hospital, Nanjing Medical University, Wuxi Medical School, Jiangnan University, Wuxi, China; ^10^Centre for Clinical Brain Sciences, University of Edinburgh, Edinburgh, United Kingdom; ^11^Usher Institute of Population Health Sciences and Informatics, University of Edinburgh, Edinburgh, United Kingdom; ^12^Department of Blood Transfusion, Beijing Tiantan Hospital, Capital Medical University, Beijing, China; ^13^Department of Neurosurgery, Beijing ChaoYang Hospital, Capital Medical University, Beijing, China

**Keywords:** severe spontaneous intracerebral hemorrhage, antiplatelet therapy, postoperative management, major cardiovascular/cerebrovascular and peripheral vascular events, randomized controlled trial

## Abstract

**Background:**

For severe spontaneous intracerebral hemorrhage (sSICH) patients with high risk of ischemic events, the incidence of postoperative major cardiovascular/cerebrovascular and peripheral vascular events (MACCPE) is notable. Although antiplatelet therapy is a potential way to benefit these patients, the severe hemorrhagic complications, e.g., intracranial re-hemorrhage, is a barrier for early starting antiplatelet therapy.

**Objectives:**

This randomized controlled trial aims to identify the benefit and safety of early starting antiplatelet therapy after operation for sSICH patients with high risk of ischemic events.

**Methods:**

This study is a multicenter, prospective, randomized, open-label, blinded-endpoint trial. We will enroll 250 sSICH patients with a high risk of ischemic events (including cerebral infarcts, transient ischemic attack, myocardial infarction, pulmonary embolism, and deep venous thrombosis). The participants will be randomized in a 1:1 manner to early-start group (start antiplatelet therapy at 3 days after operation) and normal-start group (start antiplatelet therapy at 30 days after operation). The early-start group will receive aspirin 100 mg daily. The control group will not receive antithrombotic therapy until 30 days after operation. The efficacy endpoint is the incidence of MACCPE, and the safety endpoint is the incidence of intracranial re-hemorrhage.

**Discussion:**

The Early-Start antiplatelet therapy after operation in patients with spontaneous intracerebral hemorrhage trial (E-start) is the first randomized trial about early start antiplatelet therapy for operated sSICH patients with a high risk of ischemic events. This study will provide a new strategy and evidence for postoperative management in the future.

**Clinical trial registration:**

ClinicalTrials.gov, identifier NCT04820972; Available at: https://clinicaltrials.gov/ct2/show/NCT04820972?term=NCT04820972&draw=2&rank=1.

Chinese Clinical Trial Registry, identifier ChiCTR2100044560; Available at: http://www.chictr.org.cn/showproj.aspx?proj=123277.

## Introduction

Severe spontaneous intracerebral hemorrhage (sSICH), characterized by a massive intracerebral hemorrhage and coma, is the most acute subtype of hemorrhagic stroke, with considerable disability and mortality (Cordonnier et al., 2018; Gross et al., 2019). Notably, sSICH patients are characterized by old age and usually have ischemic-arterial-related comorbidities (Wu et al., 2021). Although surgical treatment can improve the outcome and reduce the mortality of sSICH patients (Luzzi et al., 2019; Wu et al., 2021), they are still threatened by the risk of ischemic artery events postoperatively, e.g., major cardiovascular/cerebrovascular/peripheral vascular events (MACCPE; Kumar et al., 2010; Devereaux and Sessler, 2015; Smilowitz and Berger, 2016; Goshgarian and Gorelick, 2017; Smilowitz et al., 2017; Smilowitz and Berger, 2020).

In high-and middle-income countries, about a quarter of spontaneous intracerebral hemorrhage patients received antiplatelet therapy before hemorrhage (Lovelock et al., 2007). Moreover, for patients suffering from spontaneous intracerebral hemorrhage, ischemic artery events are the important factor related to the long-term poor outcome (Flynn et al., 2010; Pennlert et al., 2014; Poon et al., 2014; Casolla et al., 2019; Parasram et al., 2022). However, after hemorrhage, discontinuation of antiplatelet therapy, abnormal cardia-cerebrovascular hemodynamics and bedridden condition will certainly increase the risk of MACCPE (Devereaux and Sessler, 2015; Smilowitz and Berger, 2016; Goshgarian and Gorelick, 2017; Smilowitz et al., 2017; Ding et al., 2019; Smilowitz and Berger, 2020). Our previous study showed that approximately 20% sSICH patients had a history of antiplatelet therapy and ischemic artery events occurred in more 10% patients (Wu et al., 2021). The incident rate of MACCPE may be higher in the sSICH patients with high risk of ischemic artery events. Thus, timely and appropriate antiplatelet therapy may helpful to prevent the ischemic events and improve the outcome of sSICH patients.

Some studies reported that antiplatelet therapy might be an effective way to prevent MACCPE (Tran and Anand, 2004; Ding et al., 2018). However, the status quo is that there were just a few studies and clinical trials for perioperative management of antiplatelet therapy. Thus, no reliable protocol has been promoted to indicate when and how a sSICH patient could receive the antiplatelet therapy after appropriate surgical treatment. Considering the ischemic artery events usually occurred within 1 month after hemorrhage (Murthy et al., 2020), we assume that early antiplatelet therapy can prevent sSICH patients from MACCPE.

Here, we prepare to conduct a randomized controlled trial, aiming to investigate the efficacy and safety of early starting antiplatelet therapy in sSICH patients with high risk of ischemic artery events, postoperatively. This study will provide an evidence-based medical basis for postoperative management of antiplatelet therapy.

## Materials and methods

### Study design

This study is a multicenter, prospective, randomized, open-label, blinded-endpoint trial, which begins in May 2021, and is scheduled to be completed by May 2023. After operation, sSICH patients with high risk of ischemic events will be randomized in a 1:1 manner to early start group (start antiplatelet therapy at postoperative 3 days) and normal start group (start antiplatelet therapy at postoperative 30 days). The primary efficacy endpoint is the incident of MACCPE, and safety endpoint is the incident of re-hemorrhage. This randomized controlled trial (RCT) will follow the Consolidated Standards of Reporting Trials (CONSORT) guidelines.

### Patient selection

The inclusive/exclusive criteria are shown in Table 1. Patients suffering from sSICH, aged 18~70 years, will be screened after operation. Firstly, we will exclude patients (1) with brain tumors and cerebrovascular diseases (e.g., arteriovenous malformation and intracranial aneurysm); (2) with a history of salicylic acid allergy; (3) with hemorrhagic transformation subtype of stroke; (4) with secondary hemorrhage of venous embolism; (5) with malignant tumors; (6) receiving antithrombic therapy regardless of antiplatelet therapy; (7) with thrombocytopenia or coagulopathy disorders, and (8) with a history of atrial fibrillation. The reminded patients will receive an evaluation to identify the serum total cholesterol level, HDL cholesterol before randomization. The sSICH patients with the high postoperative risk of ischemic artery events will be enrolled into a according to follow criteria: (1) patients have a history of cerebral infarction or transient ischemic attack (TIA); (2) patients have a history of coronary heart disease or myocardial infarction; (3) ASCVD Risk Estimator Plus (Muntner et al., 2014; Cook and Ridker, 2016; Lloyd-Jones et al., 2019; mainly consider age, sex, race, systolic blood pressure, diastolic blood pressure, total cholesterol, HDL cholesterol, history of diabetes, smoker, on hypertension treatment): 10-year risk > 10% (Society CS, 2016); (4) The Caprini Risk Scale > 2 (Caprini et al., 1991; Obi et al., 2015). Patients should meet any one of the first three criteria and meet the fourth criteria simultaneously. If a sSICH patient have a new occurrence of cerebral infarction or intracerebral hemorrhage, venous thrombus embolism or acute coronary syndrome within 3 days after operation, they will be not considered in subsequent randomization.

**Table 1:** The inclusive/exclusive criteria.

**Inclusion criteria** Aged 18–70 years^*^ Nontraumatic spontaneous intracerebral hemorrhage^*^ Patients with postoperative high risk of MACCPE^*^ Previous history of cerebral infarction or TIA. Previous history of coronary heart disease or myocardial infarction. ASCVD Risk Estimator Plus: 10 years risk > 10%. The Caprini Risk Scale: Score > 2. Received neurosurgical procedures Signed informed consent. No history of allergy to salicylic acid preparation.^*^ Complete the preintervention assessment and meet the fellow criteria: Postoperative head CT showed no new infarction or hemorrhage Postoperative venous ultrasound of the lower extremity did not reveal deep vein thrombosis Postoperative electrocardiogram and myocardial enzyme examination did not show acute myocardial ischemia or myocardial infarction.
**Exclusion criteria** Structural cerebrovascular lesions (such as intracranial aneurysms, cerebrovascular malformations, etc.) or tumors in the area of bleeding or the bleeding is suspected to be related to these lesions Ischemic stroke with hemorrhagic conversion Secondary bleeding due to venous embolism The malignant tumor is expected to have a survival of no more than 3 months. Take antithrombotic agents, such as Vitamin K antagonists (warfarin) or new anticoagulants (Dabigatun Rivaroxaban) in addition to antiplatelet agents. Previous history of thrombocytopenia or coagulation disorders. Previous history of atrial fibrillation. Antiplatelet-related intracerebral hemorrhage Cerebral amyloid angiopathy-related intracerebral hemorrhage

* The inclusion criteria of primary screening.

### Endpoints and relative definitions

The primary efficacy endpoint is the incidence of MACCPE after operation. The MACCPE event is identified when patients suffer from a new cerebral infarct outside the surgical area, enlarging cerebral infarction area, new-onset TIA, more frequent TIA, new-onset myocardial infarction, reperfusion therapy for original myocardial infarction, pulmonary embolism, or deep venous thrombosis.

The other outcomes included: (1) the 3-month mortality after surgery; (2) in-hospital mortality; (3) 30- and 90-day neurological function after operation, which is assessed using the modified Rankin scale (van Swieten et al., 1988) (mRS) or Glasgow outcome scale (Jennett and Bond, 1975; GOS).

The safety endpoint is the incidence of hemorrhagic complications during antiplatelet therapy, i.e., the radiological re-hemorrhage of the operative region (comparing with the preoperative or postoperative first images) or new occurrence of intracerebral hemorrhage, subdural hemorrhage, subarachnoid hemorrhage or intraventricular hemorrhage.

For the cerebral infarcts, immediate CT (due to sudden or continuously deteriorating neurological state) and regular follow-up CT (postoperative 7 ± 5 days, postoperative 90 ± 10 days) will screen new hypodense lesions. If new hypodense lesions are found, an MRI will be used to determine the lesions. New cerebral infarcts outside the surgical area were defined as new hypodense lesions on T2-weighted MRI, which are visible in at least two continuous views. Enlarging infarctions were defined as an increase in a previously existing area of T2-weighted signal hyperintensity of at least 3 mm in one dimension (Vendt et al., 2009).

Transient ischemic attack (TIA) was defined as a transient episode (duration of symptoms < 24 h) of neurological dysfunction caused by focal brain, spinal cord, or retinal ischemia without showing acute infarction on CT images. The primary endpoint was met if patients presented with new-onset TIA or more frequent TIA.

Myocardial infarction is defined as myocardial necrosis in a clinical setting consistent with myocardial ischemia. These conditions can be satisfied by a rise of cardiac biomarkers (preferably cardiac troponin [cTn]) plus at least one of the following: (1) symptoms of cardiovascular ischemia; (2) ECG changes indicative of new ischemia (significant ST/T changes or left bundle branch block); (3) development of pathologic Q waves; (4) imaging evidence of new loss of myocardium or new regional wall motion abnormality; (5) angiography or autopsy evidence of intracoronary thrombus.

The patients who had CT pulmonary angiography or autopsy evidence of pulmonary embolism were defined as pulmonary embolism. For the patients who had no evidence, if they fulfilled the criteria described below, they were also defined as pulmonary embolism. The criteria include (1) clinical manifestations of pulmonary embolism: cough, chest pain, hemoptysis, dyspnea, etc.; (2) Wells score > 4; (3) blood tests showed high D-dimer levels, high Troponin levels, low blood oxygen saturation, high alveolar-arterial (A-a) oxygen partial pressure; (4) venous ultrasound of lower extremities showed deep vein thrombosis.

Deep venous thrombosis was defined as the follow-up venous ultrasound of lower extremities that showed hyperechoic or mixed echogenicity in deep venous. Deep veins mainly include femoral vein, popliteal vein, anterior tibial vein, posterior tibial vein, fibular vein, and iliac vein.

Re-hemorrhage of the operative region was defined as new high-density focal lesions in the surgical area or original hematoma enlargement comparing with the preoperative CT and first postoperative CT. Postoperative subdural hemorrhage, subarachnoid hemorrhage, and intraventricular hemorrhage were also confirmed by postoperative CT.

Two investigators will identify blinded to the patient information assesses the occurrence of MACCPE and rehemorrhage events on 10 ± 4 and 90 ± 14 days post-surgery. mRS scores, GCS, and GOS will be assessed on 30 days/discharge and 90 ± 14 days post-surgery.

### Procedure and data collection

Data collection includes demographic information, clinical information, imaging information, and laboratory examinations. The details are shown in Table 2.

**Table 2:** Information collection.

Information collection	Content
Clinical information	Demographic information, health economics information, physical examination, etc.
Surgery information	Type of surgical treatment, blood information, hemostasis subjective difficulty score, etc.
Clinical score	mRS score, GCS, ICH score, GOS
Special examinations of MACCPE	Head CT, venous ultrasound of lower extremities, myocardial enzyme, thromboelastogram
Others laboratory examinations	Blood routine, biochemistry, coagulation function
Outpatient or phone review	Rebleeding event, MACCPE, survival condition

**Figure 1:**
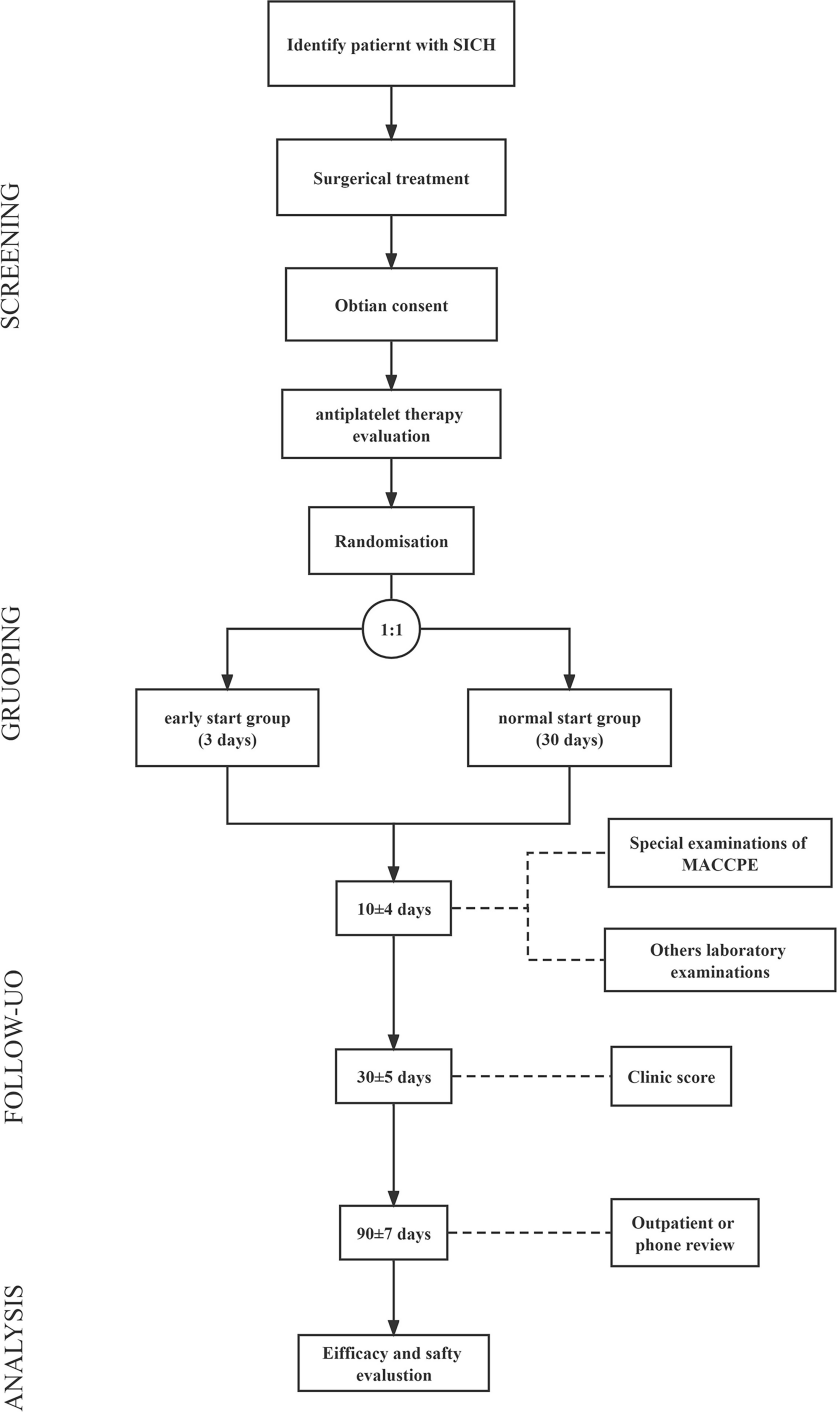
The flowchart of study procedures.

The procedure includes screening (Step 1), randomization and administration (Step 2), and outcome evaluation (Step 3; Figure 1; Table 3).

**Table 3:** Study procedures.

	STUDY PROCEDURES						
	Screening			Treatment	Follow-up		
	Admission	Intraoperative	Within 72 h	At 3 days	10 ± 4 days	30 ± 5 days	90 ± 7 days
Randomization				X			
Start antiplatelet therapy				X			
Clinical information	X						
Clinic score	X					X	X
Surgery information		X					
Special examinations of MACCPE			X		X		X^*^
Others laboratory examinations			X		X		X^*^
Outpatient or phone review							X

* The examinations will be completed on outpatient.

#### Step 1: Screening and enrollment

As shown in Figure, patients will receive a primary screening before operation. The evaluation includes head computational tomography (CT), deep venous ultrasound, coagulation and myocardial enzyme examination. The patients with (1) re-hemorrhage or residual hematoma expansion, (2) deep vein thrombosis, and (3) abnormality in myocardial enzymes examination.

### Step 2: Randomization and administration

The patients will be randomized in a 1:1 manner to the early start group and the normal start group. After informed consent, all enrolled patients will be randomly grouped using an online randomization system (Churun Information Technology Co., Guangdong, China). The patients in early start group will receive aspirin 100 mg per day. The patients in normal start group will not receive antithrombotic therapy until 30 days after operation.

#### Step 3: Outcome evaluation

During hospital stay, patients will receive a series of special examinations of MACCPE at 10 ± 4 days after administration of aspirin, in order to identify whether there is an occurrence of MACCPE. If patients have a sudden unconsciousness, or suddenly or gradually worsening neurological states after administration of aspirin, a head CT examination will be conducted immediately to identify whether there is an occurrence of re-hemorrhage. If the patient occurs ischemic events, the antiplatelet or anticoagulation therapy will be added to the patient based on the specific condition. These patients meet the endpoint when ischemic events occur, so they will not be excluded from this study.

**Figure 2:**
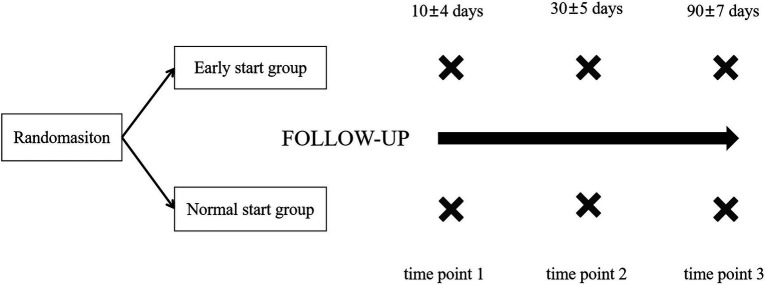
Follow-up plan.

Patients will be regularly followed up at 30 and 90 days. To the patient who did not discharge in 30 days, we would assess the clinic score at the bedside. To the discharged patient, we assess the clinic score *via* outpatient or phone review. At 90 days, if the patient could not complete the follow-up examination in the medical centers involved to the study, they will be required to complete the relative examinations in the local hospitals. Our investigators will ask patients’ family members to upload the follow-up data and assess the quality of these data. If these data are out of required quality and more details could not be got, the patients will be considered as the patients withdrawing from the follow-up (Figure 2).

During observation, investigators will detailly record the following events:

1. MACCPE (the occurrence time, special treatment and radiological data).
2. Mortality (the occurrence time and cause of death).
3. Neurological function outcome, which is evaluated by the mRS, GCS, and GOS.
4. Intracranial re-hemorrhage or intracranial bleeding potentially related to aspirin (the occurrence time, treatment, radiological data and cause of bleeding).
5. Regular follow-up head CT.

### Monitoring the effectiveness of antiplatelet therapy

Before and after aspirin administration, we will assess the platelet function. The platelet function will be assessed using the thromboelastogram (TEG). All parameters, including maximum amplitude (MA), citric acid kaolin (CK), reptilase (A), aspirin (AA), are recorded through standard tracing. Based on MA, the rate of platelet function inhibited by aspirin (AA%) was calculated using the formula as shown below:


$$AA\%=\frac{MA\left(CK\right)-MA\left(AA\right)}{MA\left(CK\right)-MA\left(A\right)}$$

For patients in the early start group, the platelet function will be detected before aspirin administration and 7 days after administration. AA% > 50 is defined as the good response to aspirin. Moreover, for the patients in the normal start group, because they do not receive any antiplatelet therapy, the platelet function assessment do not need to perform for these patients.

### Quality control of the research

To avoid participant bias and ensure a sufficient source of operated sSICH patients, we will enroll appropriate centers according to follow standards: (1) the medical centers cover at least two communities or work as a referral unit for hemorrhagic stroke; (2) the center can conduct various treatments for sSICH and can fully represent the diagnosis and treatment of sSICH at a national level; (3) the medical center treats at least 50 sSICH patients annually by surgical methods; (4) the center can complete all examinations which are required in the study. Finally, nine medical centers encounter our standards and participate in this study. This study will conduct in nine regional neurosurgical centers simultaneously.

In this study, operation decision is made based on the consensus of neurosurgeons and patient family, according to clinical guideline. The operation is performed by a senior neurosurgeon, working as a neurosurgeon for more than 10 years. After operation, all of the patients would receive standardized care according to the guidelines. Before and after operation, patient will receive blood pressure management, and the target blood pressure is <140/90 mmHg.

At statistical stage, we will review all postoperative treatments and further exclude: (1) the patients receiving non-indicated platelet transfusion or anticoagulation treatment; (2) the patients whose the postoperative blood pressure are abnormal even receiving special treatment; (3) the patients suffering from an abnormal change of blood cells, abnormal hypercoagulation or disseminated intravascular coagulation.

### Statistical analysis

#### Sample size

The sample size is estimated using the PASS 15 (PASS corporation, United States). In our preliminary study, we investigated the clinical characteristics and outcome of operated sSICH patients in Beijing Tiantan Hospital. The incidence of main adverse cardiovascular and cerebrovascular events was 10.8% after operation (Wu et al., 2021). The E-start study expects to reduce the incident of MACCE by 5%. Patients were randomized at a ratio of 1:1 to one of two groups. At (1 − β) as 0.8 and α as 0.05, 244 consecutive patients would be enrolled in this study (122 in the early start group and 122 in the normal start group). This study prepares to enroll 250 operated sSICH patients, including 125 subjects in the early start group and 125 subjects the normal start group.

##### Endpoint and outcome evaluation

We intend to check the plausibility and validity before the database is locked for analysis. We will exclude the cases that could influence the accuracy of conclusion. Patients who do not meet the requirement of quality control may be further excluded based on the consensus of investigators’ discussion.

Categorical variables are presented as numbers (no.) and percentage (%). Continuous variables with normal distribution are expressed as means and standard deviation, and medians and inter-quartile range (IQR) if otherwise. The differences between groups in continuous variables are compared by performing the student’s t-tests or Wilcoxon rank sum tests, and the differences in categorical variables are compared using the chi-square tests or Fisher’s exact tests. The incident rate (IR) of PR and its 95% confidence interval (CI) is calculated. The univariate and multivariate Cox regression analyses are performed in PR-related analysis. The result of Cox regression analysis is presented as hazard ratio (HR) and 95% CI. The statistical analyses are conducted with SPSS 24.0 (SPSS, Chicago, IL), with two-sided *p* < 0.05 considered of statistical significance.

###### Subgroups analysis

Seven subgroups analysis will be performed, including subgroup by age, subgroup by gender, subgroup by weight, subgroup by BMI, subgroup by platelet function, subgroup by ischemic lesion, and subgroup by the type of surgery. Subgroup analyses are limited to the primary efficacy endpoint and safety endpoint only. Separate Cox models will be applied to each subgroup during subgroup analysis.

#### Data management

The clinical research coordinator (CRC) will make regular visits to each participating center to ensure the strict performance of the research program. In case of any contradiction to the study protocol, the CRC will promptly report this to the investigators. In addition, throughout this study, a summary meeting will be held every 3 months to discuss and solve any problem arising from the research or encountered by the patients.

All data will be collected and stored using case report form (CRF), and the completed CRFs will be sent back to Beijing Tiantan Hospital per 3 months. In addition, the data will be also transcribed into an electronic version by nine investigators independently. After completion, the CRFs will be sealed in a research-specific cabinet, and a particular person shall keep the electronic data. The principal investigator’s institution established a data management committee in Beijing Tiantan Hospital to monitor data quality and ensure data security. Data was accessible only to the members of the research team. The privacy of the patients involved was protected.

#### Ethical considerations

This study was granted approval from the Institutional Review Board of Beijing Tiantan Hospital (KY 2021–053-02), and the research would be conducted in strict accordance with the declaration of Helsinki and Human Biomedical Research Ethical Issues. All enrolled subjects will be informed with a complete and comprehensive introduction, including the purpose, procedure, potential risks, and potential benefits of the study. The patient’s family should sign the written informed consent before beginning. They will be informed that they have the right to withdraw from the study at any time, and informed consent will be retained as a clinical study document for future reference. The subject’s personal privacy and data confidentiality will be protected during the study.

## Discussion

E-start study is the first clinical trial of postoperative early start antiplatelet therapy in sSICH patients with high risk of ischemic events. In this study, we investigate whether early start antiplatelet therapy after surgery can prevent the incident of MACCPE, with no increasing risk of postoperative intracranial rehemorrahge.

Early start antiplatelet therapy postoperatively may reduce the incidence of MACCPE that seriously affects the clinical outcome of sSICH patients (Ding et al., 2018). However, there is no standardized protocol for postoperative antiplatelet management. Therefore, we analyzed the characteristics of postoperative MACCPE incidence by the SAP-ICH cohort (ChiCTR1900024406). We find that MACCPE occurs four postoperative days later, and the peak is the seventh day after surgery. Remarkably, there were often more than 30 days before starting antiplatelet therapy postoperatively in past research (Flynn et al., 2010; Murthy et al., 2019; RESTART Collaboration, 2019). Therefore, it may be missing the time window of the high incidence of MACCPE, which may mask treatment effects. Summarily, early start antiplatelet therapy (start in postoperative 4 days) is crucial for decease the incidence of MACCPE.

Notably, safety is another consideration in this study. The data of SAP-ICH showed that intracerebral hemorrhage events often occur 3 days following the procedure. In addition, many studies have reported the results that restarting antiplatelet therapy after ICH does not increase the risk of recurrent intracerebral hemorrhage (Flynn et al., 2010; Teo et al., 2017; Chen et al., 2018; Murthy et al., 2019; RESTART Collaboration, 2019; Diep and Garcia, 2020; Al-Shahi Salman et al., 2021). The results of an observational study published in *stroke* showed that the rate of recurrent bleeding after discharge in sSICH patients treated with antiplatelet agents was roughly the same as that in patients not treated with antiplatelet agents (Flynn et al., 2010). The “RESTART” research, a randomized controlled study published in the Lancet, further confirmed that the risk of a recurrent intracerebral hemorrhage after SICH using antiplatelet therapy is small (RESTART Collaboration, 2019). However, this assumption should be verified by the result of this study.

## Conclusion

In summary, we have described the first clinical trial for early start antiplatelet therapy for operated sSICH patients with a high risk of MACCPE. The efficacy and safety of early start antiplatelet therapy will be discussed in this study, which will provide evidences for postoperative antithrombotic management in the future.

## Ethics statement

The studies involving human participants were reviewed and approved by IRB of Beijing Tiantan Hospital, Capital Medical University. The patients/participants provided their written informed consent to participate in this study.

## Author contributions

SW is the principal investigator of this study and obtained the research funding. KW, SM, QL, JW, and SW have developed this study protocol. KW, SM, and QL are the main author of this article and contributed equally. KW, SM, QL, JW, RS, and SW revised this manuscript. JP, XH, DK, FL, XL, DZ, XS, JR, XT, JL, and KW are the main people responsible for the nine clinical centers and responsible for implementing this study. KW, QL, JW, YZ, and NW provided statistical and technical supports. SW, JW, and XL approved publication of this final manuscript. SG and YL are responsible for recruitment of patients. The authors read and approved the final manuscript. All authors contributed to the article and approved the submitted version.

## Funding

National Key Research and Development Program of the 14th Five-Year Plan (grant no. 2021YFC2501100).

## Conflict of interest

The authors declare that the research was conducted in the absence of any commercial or financial relationships that could be construed as a potential conflict of interest.

## Publisher’s note

All claims expressed in this article are solely those of the authors and do not necessarily represent those of their affiliated organizations, or those of the publisher, the editors and the reviewers. Any product that may be evaluated in this article, or claim that may be made by its manufacturer, is not guaranteed or endorsed by the publisher.
